# Delving into defence: identifying the *Pseudomonas protegens* Pf-5 gene suite involved in defence against secreted products of fungal, oomycete and bacterial rhizosphere competitors

**DOI:** 10.1099/mgen.0.000671

**Published:** 2021-11-17

**Authors:** Silas H. W. Vick, Belinda K. Fabian, Catherine J. Dawson, Christie Foster, Amy Asher, Karl A. Hassan, David J. Midgley, Ian T. Paulsen, Sasha G. Tetu

**Affiliations:** ^1^​ Department of Molecular Sciences, Macquarie University, North Ryde, Australia; ^2^​ Commonwealth Scientific and Industrial Research Organisation (CSIRO), North Ryde, Australia; ^3^​ Faculty of Chemistry, Biotechnology and Food Science, Norwegian University of Life Sciences, Ås, Norway; ^4^​ ARC Centre of Excellence in Synthetic Biology, Macquarie University, North Ryde, Australia; ^5^​ School of Environmental and Life Sciences, University of Newcastle, Newcastle, Australia

**Keywords:** biocontrol, competition, interkingdom interactions, PGPR, rhizosphere, saturation mutagenesis

## Abstract

Competitive behaviours of plant growth promoting rhizobacteria (PGPR) are integral to their ability to colonize and persist on plant roots and outcompete phytopathogenic fungi, oomycetes and bacteria. PGPR engage in a range of antagonistic behaviours that have been studied in detail, such as the production and secretion of compounds inhibitory to other microbes. In contrast, their defensive activities that enable them to tolerate exposure to inhibitory compounds produced by their neighbours are less well understood. In this study, the genes involved in the *Pseudomonas protegens* Pf-5 response to metabolites from eight diverse rhizosphere competitor organisms, *Fusarium oxysporum*, *Rhizoctonia solani*, *Gaeumannomyces graminis* var. *tritici*, *Pythium spinosum*, *Bacillus subtilis* QST713, *Pseudomonas* sp. Q2-87, *Streptomyces griseus* and *Streptomyces bikiniensis* subspecies *bikiniensi*, were examined. Proximity induced excreted metabolite responses were confirmed for Pf-5 with all partner organisms through HPLC before culturing a dense Pf-5 transposon mutant library adjacent to each of these microbes. This was followed by transposon-directed insertion site sequencing (TraDIS), which identified genes that influence Pf-5 fitness during these competitive interactions. A set of 148 genes was identified that were associated with increased fitness during competition, including cell surface modification, electron transport, nucleotide metabolism, as well as regulatory genes. In addition, 51 genes were identified for which loss of function resulted in fitness gains during competition. These included genes involved in flagella biosynthesis and cell division. Considerable overlap was observed in the set of genes observed to provide a fitness benefit during competition with all eight test organisms, indicating commonalities in the competitive response to phylogenetically diverse micro-organisms and providing new insight into competitive processes likely to take place in the rhizosphere.

## Data Summary

Sequence data from TraDIS (transposon-directed insertion site sequencing) are available from the European Nucleotide Archive. Sequence files for the treatments are available under the project accession number PRJEB39220 and sample accession numbers ERS4802861 to ERS4802876. Sequence files for control samples are available under the project accession number PRJEB46191 and sample accession numbers ERS6644756 and ERS6644757.

**Impact Statement**

Microbial life involves interactions with diverse neighbouring microbial cells. These interactions are often antagonistic, where multiple microbes compete for limited resources. Whilst these antagonistic interactions have been known about and widely studied, it has often been from the perspective of the aggressor, and research has focused heavily on the production of antagonistic secondary metabolites and other factors for the inhibition or killing of neighbouring cells. In contrast, there has been less focus on the defensive mechanisms microbes have devised to tolerate and survive these interactions. Here, transposon-directed insertion site sequencing (TraDIS) has been used to assay the role of each gene in the genome of *Pseudomonas protegens* Pf-5 in protecting against the secreted metabolites of eight diverse rhizosphere competitor organisms. The results of this study have identified a broad spectrum of genes, many of which have not before been identified as playing a role in defensive capabilities. These findings are relevant to the field of plant growth promoting and biocontrol bacteria, contributing to our understanding of the antagonistic interactions these biocontrol bacteria undergo with the phytopathogenic micro-organisms they are aimed at controlling.

## Introduction

The soil and particularly the rhizosphere of plants are populated by an extremely diverse array of microbes including fungi, protists, oomycetes, phage, bacteria and archaea. To survive in this densely populated environment, these microbes have evolved numerous mechanisms to enhance competitive fitness. These include both competitive nutrient acquisition and metabolic flexibility as well as antagonistic behaviours, such as the production and secretion of toxic secondary metabolites, peptides and exoenzymes that directly inhibit the growth of surrounding micro-organisms [1–3]. The evolution of these antimicrobials secreted by micro-organisms in soil and the rhizosphere required the parallel development of cellular defences by cohabiting microbes as well as the producing microbes themselves [4, 5]. Mechanisms of cellular defence, while likely key to successful rhizosphere colonization and persistence, have not yet been widely explored at a genetic level.

Plant growth promoting rhizobacteria (PGPR) are often involved in suppression of phytopathogens and, thus, have potential as biocontrol agents for the prevention of crop diseases. To effectively deploy these bacteria as biocontrol agents requires detailed knowledge of the ways in which they compete with and protect themselves from other organisms in the rhizosphere niche, particularly phytopathogens. For many biocontrol bacteria, there is now detailed knowledge of the genetic basis for the arsenal of compounds they produce and secrete during antagonistic interactions, but less is known regarding the structures and mechanisms by which they defend themselves against compounds produced by their neighbours. Improving our understanding of this aspect of rhizosphere interactions would facilitate efforts to determine when and where biocontrol bacteria might be applied most efficaciously. Furthermore, since most of the currently used classes of antibiotics are based on secreted antimicrobial secondary metabolites produced by soil-dwelling microbes, detailed knowledge of these defences will provide unique insight into development of antibiotic resistance in bacterial relatives of PGPR that have become opportunistic human pathogens since our widespread use of antibiotics began.

Several species of PGPR pseudomonads are known to be successful biocontrol agents of plant pathogens. *Pseudomonas* are ubiquitous Gammaproteobacteria that have adapted to survive and proliferate in a wide range of environments, including those associated with host organisms, through both positive and negative symbioses. PGPR pseudomonads have developed cooperative symbiotic relationships with plant hosts [6]. The plants provide nutrients for the bacteria, through root exudates, as well as surfaces on which to grow and, in return, PGPR pseudomonads can positively affect plant health by enhancing access to nutrients [7, 8], production of phytohormones [9–12], and suppression of pests and phytopathogenic micro-organisms including bacteria, fungi and oomycetes [13]. These beneficial effects on plant growth and health have led to the use of certain commensal pseudomonads as commercial biocontrol agents, used to suppress a range of phytopathogens [14].

*Pseudomonas protegens* Pf-5 is a plant commensal pseudomonad that has been demonstrated to protect agriculturally important plants, including cotton [15, 16] and cucumber [17], from fungal and oomycete phytopathogen infection. Traits leading to successful biocontrol by Pf-5 include attachment to root surfaces, proliferation using plant exudates as nutrient sources, induction of systemic resistance in the plant [18, 19], and the production of antimicrobial secondary metabolites inhibitory to phytopathogenic micro-organisms [15–17, 20–22]. Here, we have set out to investigate the less-well understood mechanisms of how Pf-5 defends itself against the plethora of antimicrobial compounds produced by soil competitors and identify the genes required for such defence.

Genome-wide saturation mutagenesis and transposon-directed insertion site sequencing (TraDIS) are powerful techniques for the study of genes necessary for survival under conditions where an array of genes are likely to contribute to fitness [23]. The large size of pseudomonad genomes and their considerable plasticity has meant that many genes remain functionally uncharacterized, and our understanding of genes involved in rhizosphere interactions is still relatively limited [12]. Therefore, TraDIS is an ideal technology to apply to questions of host defence mechanisms in Pf-5. In this work, a saturated *Pseudomonas protegens* Pf-5 transposon mutant library was grown adjacent to eight different rhizosphere micro-organisms, three fungal and one oomycete phytopathogen and four bacterial rhizosphere competitors, to identify genes involved in cellular defence against varied fungal, oomycete and bacterial secondary metabolites.

## Methods

### Media and strains

Gnotobiotic cultures of *Pseudomonas protegens* Pf-5 and other rhizosphere organisms were established on King's medium B (KB) agar plates (20 g proteose peptone l^−1^, 1.5 g K_2_HPO_4_ l^−1^, 1.5 g MgSO_4_ .7H_2_O l^−1^, 15 ml glycerol l^−1^, 20 g agar l^−1^, final pH of 7.2) and incubated at room temperature [24]. Rhizosphere micro-organisms used in the current study were *Pythium spinosum* P.46.1, *Gaeumannomyces graminis* var. *tritici* GV1 (CSIRO culture collection), *Fusarium oxysporum* (CSIRO culture collection), *Rhizoctonia solani* PM1 (CSIRO culture collection), *Bacillus subtilis* QST713 (Serenade; Bayer AgroSciences), *Pseudomonas* sp. Q2-87 [12], *Streptomyces bikiniensis* subspecies *bikiniensi* ATCC 11062 and *Streptomyces griseus* NRRL B-2165.

### Examination of secondary metabolite production by fungal and oomycete competitors

Fungi and oomycetes were plated on KB agar plates in duplicate and grown at room temperature for 5 days. A suspension of Pf-5 was added to these plates in eight 6 mm diameter holes cored in the agar plates in a ring around the region of fungal growth and plates were incubated at room temperature (22 °C) for 3 days (Fig. S1a available in the online version of this article). After incubation, triplicate 6 mm diameter agar plugs were cut from the outer region of fungal growth, 500 µl methanol:dichloromethane:ethylacetate (1 : 2 : 3) was added and left for 48 h at room temperature. Plugs in solvent were sonicated on high for 60 min (Elmasonic S60H). The majority of residual solvent was pipetted off the agar plugs and the remainder removed under a purified nitrogen stream and replaced with 500 µl 100 % methanol. Extracts were transferred to 1.5 ml Eppendorf tubes and centrifuged at 1000 ***g*** for 5 min to remove any debris before analysis with HPLC. Axenic cultures for comparisons were grown under the same conditions and for the same periods of time without the addition of a partner, and extractions of these were carried out following the same methodology.

### Examination of secondary metabolite production by bacterial competitors

Bacteria used in competition experiments with Pf-5 were picked from colonies grown on KB agar plates and inoculated onto the centre of fresh KB agar plates using a sterile inoculation loop, spread out in a ~3 cm patch on the centre of the plate and incubated at room temperature (22 °C) for 4 days. A suspension of Pf-5 was added to these plates in eight 6 mm diameter holes cored in the agar plates in a ring around the region of bacterial growth (Fig. S1a). These cultures were incubated at room temperature for a further 4 days before agar cores were taken for solvent extraction as described above for fungi. Agar cores were added to 500 µl methanol, sonicated for 60 min on high (Elmasonic S60H), then left for 48 h at room temperature. Solvent was transferred to 1.5 ml Eppendorf tubes, and centrifuged at 1000 ***g*** for 5 min to remove debris before analysis with HPLC. Axenic cultures for comparisons were grown under the same conditions and for the same periods of time without the addition of a partner, and extractions of these were carried out following the same methodology.

### HPLC analysis

HPLC analysis was performed on a 1290 Infinity II HPLC system (Agilent) with a 1290 Infinity diode-array detector collecting spectra at 205 nm with a bandwidth of 4 nm. Separations were done on a ZORBAX StableBond C18, 2.1×50 mm, 1.8 µm column (Agilent). Elution was carried out with a flow rate of 0.5 ml min^−1^ using a gradient of 10–100 % acetonitrile/H_2_O (+0.01 % trifluoroacetic acid) over 17 min.

### Transposon mutant library construction

A previously constructed Pf-5 transposon mutant library was used in this study [25]. Briefly, a kanamycin (Km)-resistant Tn*5* transposome was constructed using EZ-Tn*5* transposase (EpiCentre) and a transposon carrying a Km resistance cassette isolated from the plasmid pUT_Km [26]. The transposome was electroporated into freshly prepared electrocompetent Pf-5 cells, and the cells were plated onto LB-Km agar (16 μg ml^−1^). A total of ~500 000 colonies from four independent transformations were collected to create the saturated mutant library pool. Sequencing and analysis using the Bio-Tradis pipeline [27] indicated the library contains ~256 000 unique transposon insertion sites with transposon insertions spread throughout the genome, with a mean of one transposon insertion every ~27 bp equating to ~45 transposon insertion sites per non-essential gene [25].

### TraDIS library competitor challenge experiments and sequencing

All TraDIS competitor challenge experiments were performed on five replicate KB agar plates (diameter 90 mm) with growth at room temperature (22 °C). Due to the differing speed of growth of the competitors, as well as differences in Pf-5 growth with some partners, the growth periods varied for the different organisms. For uniformity between the experiments, all competitors (bacterial, oomycete and fungal) were grown on agar plates until a colony with diameter ~4–5 cm was obtained (fungi/oomycete) or a suitable density of bacterial culture achieved (*Pythium spinosum* 10 days; *R. solani* 5 days; *F. oxysporum* 6 days; *G. graminis* var. *tritici* 12 days; *B. subtilis* 3 days; *Pseudomonas* sp. Q2-87; *Streptomyces bikiniensis* subspecies *bikiniensi* and *Streptomyces griseus* 4 days). Eight 6 mm diameter holes were bored in the agar plates around the margin of growth and 20 µl overnight WT Pf-5 culture added to each hole to stimulate a response in competitor organisms (Fig. S1b). These cultures were grown for 4 days, with the exception of the *Pythium spinosum* challenge experiment, which was grown for 5 days, to induce a secondary metabolite response towards Pf-5. An aliquot of the saturated Pf-5 TraDIS library (1.09×10^9^ cells in 100 µl total volume) was then added to each plate in a ~1 cm wide ring around the competitor colony as close as possible without mixing. The Pf-5 transposon mutant library cells were left to grow for 2 (*R. solani*, *G. graminis* var. *tritici* and *Pythium spinosum*), 3 (all bacteria) or 4 days (*F. oxysporum*) until observably thick growth had formed (growth of Pf-5 transposon mutant library cells was markedly slower on plates with *F. oxysporum*). The Pf-5 transposon mutant library cells were then scraped off the agar plates using a sterile scalpel blade and frozen at −20 °C until DNA extraction and sequencing. For the controls, 100 µl of the transposon mutant library (1.09×10^9^ cells) was spread in a ~1 cm wide ring on three KB agar plates in triplicate (i.e. a total of nine plates). After 48 h at room temperature (22 °C), the ring of cells was scraped off the agar surface using a sterile plastic loop and collected in PBS.

DNA extraction of TraDIS library cells was performed using the DNeasy blood and tissue DNA extraction kit (Qiagen) with a modified protocol to improve cell lysis. Briefly, a cell lysis buffer consisting of 480 µl 100 mM EDTA (pH 8) and 120 µl (10 mg ml^−1^) lysozyme in TE was added to 20 µl of Pf-5 transposon mutant library cells and incubated at 37 °C for 3.5 h. Twenty microlitres of proteinase K solution (600 mAU ml^−1^) was then added and incubated at 56 °C overnight. The lysed cells were then processed according to the manufacturer’s bacterial DNA extraction protocol (Qiagen). DNA extracts from the four replicates with highest DNA yield and quality were taken and divided into two pools before being sent to the Ramaciotti Centre for Genomics (Australia) for TraDIS sequencing (https://www.ramaciotti.unsw.edu.au/). As the reproducibility of transposon-insertion sequencing has been shown to be extremely high, only two replicates were sequenced for each competitor interaction [28]. A linear regression of the gene insertion indexes of the replicates was completed in R [29] and showed that correlation coefficients between the insertion indexes for all pairs of replicates were >0.87 (*P*<0.01; Fig. S2), which validates the reproducibility of our replicates and is consistent with the reproducibility of transposon insertion sequencing replicates in other studies [28]. TraDIS was performed with an Illumina MiSeq platform to obtain 52 bp single-end genomic DNA reads as described elsewhere [27].

### Sequence data analysis

Sequencing reads were analysed with the Bio-Tradis bioinformatics pipeline [27]. We used the same Bio-Tradis pipeline parameters as Fabian and colleagues [25], including allowing a 1 bp mismatch in the transposon tag, excluding transposon insertions in the extreme 3′ end of each gene, and mapping reads with more than one mapping location to a random matching location. After matching to the transposon tag, between 1 072 535 and 3 267 865 reads per replicate were mapped to the Pf-5 genome (Table S1). The Tradis_comparison.R script was used to compare the read coverage of the control pool relative to the output pools and genes with log_2_-fold change greater than two and a q-score lower than 0.01 were used for further analysis [30–32]. The control pool consisted of the transposon mutant library grown on KB agar without any treatment, and the output pool consisted of the transposon mutant library after treatment where selective pressures have enriched or depleted individual mutants.

### Growth curves of Pf-5 mutation strains grown on competitor spent media

Competitors were inoculated into 5 ml KB in triplicate and grown statically at room temperature (*F. oxysporum* 6 days, Pf-5 4 days). Spent media was collected by centrifugation at 10 000 ***g*** for 10 min at room temperature, the supernatant was then filtered through a 0.22 µm syringe filter. A 96-well plate was set up with wells containing 40 % (v/v) competitor spent media and fresh KB controls (four technical replicates for each biological replicate and a control). Pf-5 WT and mutation strains were grown to mid-exponential phase in KB then used to inoculate multiwell growth plates at 5×10^5^ c.f.u. ml^−1^. Plates were incubated in a FLUOstar plate reader (BMG Labtech) at 30 °C with shaking for 20 h, with OD_600_ readings taken every 7 min.

## Results and Discussion

Research into the genetic basis of competition in biocontrol bacteria has so far focussed heavily on the biosynthetic gene clusters needed to produce secondary metabolites inhibitory to phytopathogenic micro-organisms. Here, a *Pseudomonas protegens* Pf-5 transposon mutant library was cultured adjacent to a suite of different rhizosphere micro-organisms to identify the genes used by Pf-5 to interact with and respond to the secondary metabolites and other secreted products from such rhizosphere competitors, including phytopathogenic fungi/oomycetes, soil-colonizing bacteria and other rhizospheric biocontrol bacteria.

### Phenotypic responses on exposure to competitor metabolites

To first establish that *Pseudomonas protegens* Pf-5 interacts with and responds to secreted products from the selected competitor strains, the growth of each organism was observed during adjacent cultivation on solid media plates and compared with independently grown controls, and HPLC analysis used to look for changes in secondary metabolite production. Differences in the growth of Pf-5 were observed when this strain was cultured adjacent to competitor organisms on solid media, including changes in growth rate and pigment production. The addition of Pf-5 also resulted in observable differences in the growth of the competitor organisms, including reduced growth, pigment production and changes in hyphal appearance for fungi (Fig. S3). HPLC analysis performed on agar plugs from these competing cultures and each strain grown axenically indicated culturing in proximity changed the profile of secondary metabolites in either Pf-5 or the partners compared to axenic growth [Figs S4 (bacteria) and S5 (fungi and oomycete)]. Changes included both larger peak areas, indicating production of increased amounts of a secondary metabolite under adjacent growth conditions, as well appearance of additional peaks, suggesting production of additional metabolites. In some instances, HPLC results were indicative that there was also a reduction in or loss of a Pf-5 metabolite during growth with some partners, based on missing peaks in profiles from cultures grown adjacent to competitors compared to independently grown cultures (an example from growth alongside *F. oxysporum* is indicated in Fig. S5).

### Identifying genes contributing to fitness during growth in the presence of competitors

**Fig. 1.**
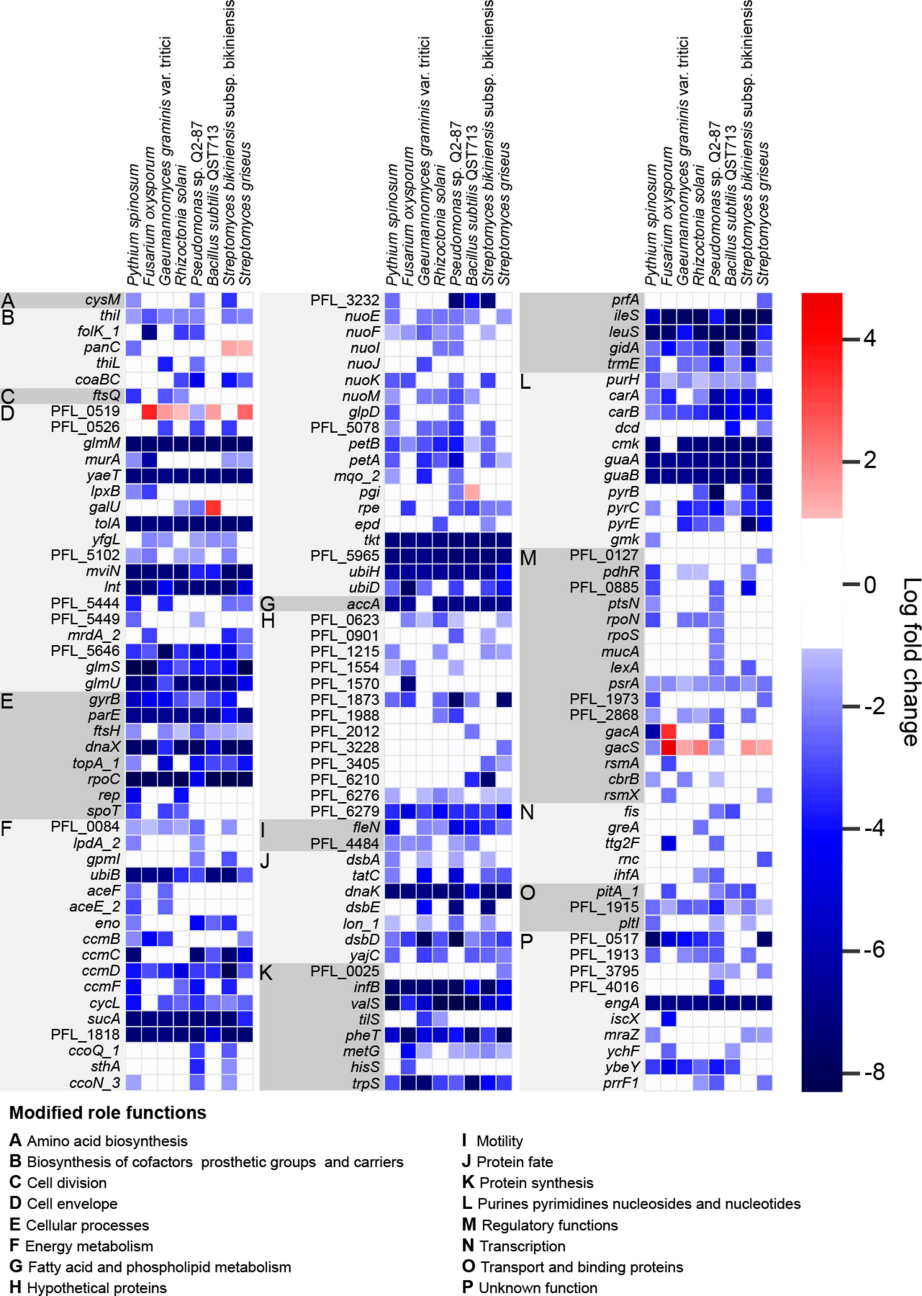
Heatmap showing *Pseudomonas protegens* Pf-5 gene mutants identified by TraDIS analysis with a >2 log_2_ fold depletion after growth in proximity with at least one of the tested competitor rhizosphere organisms, indicating genes for which loss of function was associated with reduced fitness in the presence of competitor exudates.

**Fig. 2.**
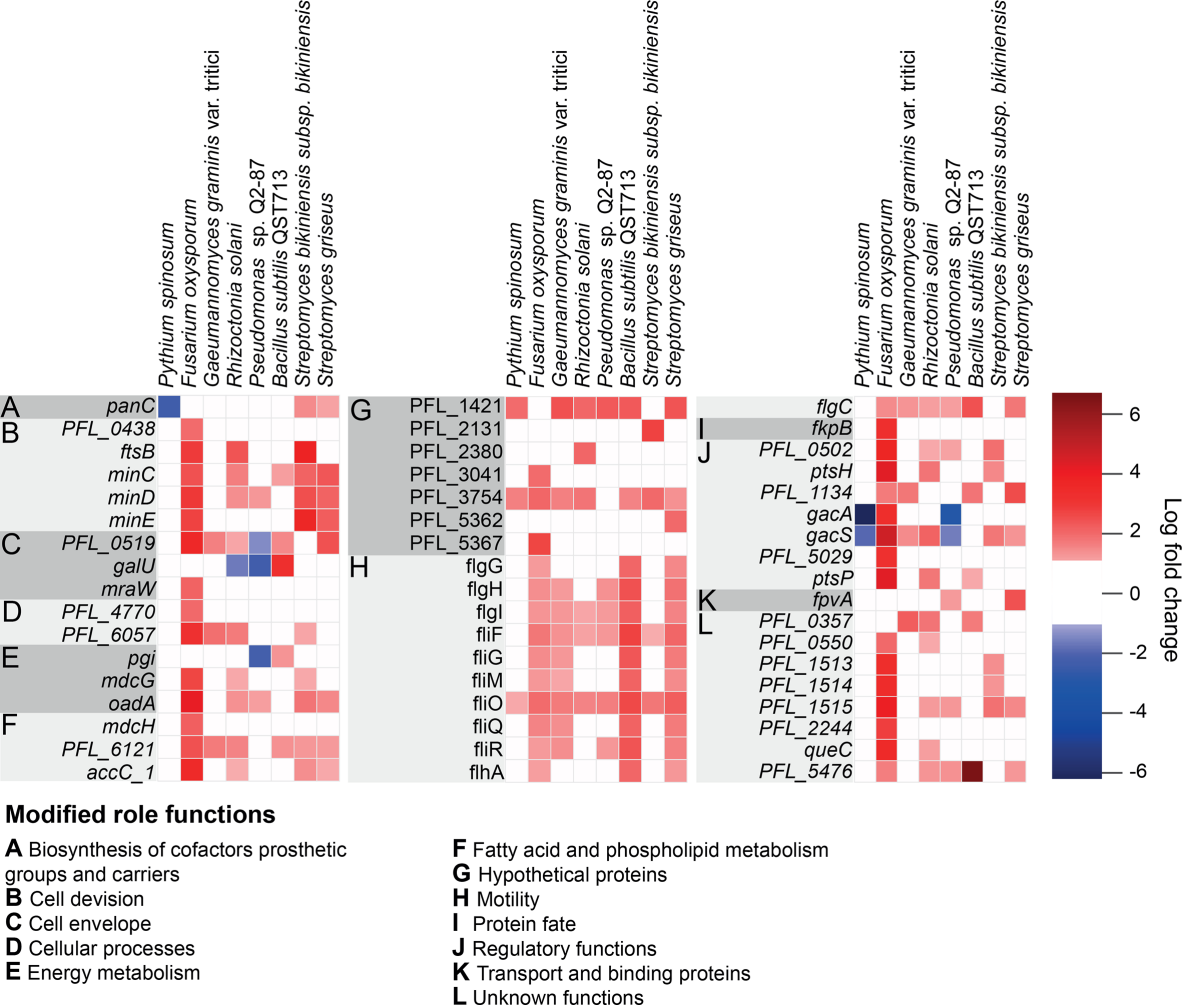
Heatmap showing *Pseudomonas protegens* Pf-5 gene mutants identified by TraDIS analysis with a >2 log_2_ fold enrichment after growth in proximity with at least one of the tested competitor rhizosphere organisms, indicating genes for which loss of function was associated with enhanced fitness in the presence of competitor exudates.

Competitor challenge experiments with the Pf-5 saturated mutant library identified a set of 148 genes for which loss of function was strongly detrimental to Pf-5 survival and growth, based on cells with mutations in these genes being present at significantly lower levels (log_2_fold change < −2) in the TraDIS mutant pool when grown adjacent to a competitor compared to the mutant pool grown alone (control) (Fig. 1). A set of 51 genes were identified for which loss of function improved Pf-5 survival and growth in competition, with cells carrying mutations in these genes being present at significantly higher levels (log_2_fold change >2) in the population grown adjacent to competitor compared to the control population grown alone (Fig. 2). The full list of depleted and enriched gene mutants from all competition experiments is available in (Table S2).

There was considerable overlap in the set of genes for which loss of function resulted in reduced fitness during competition, i.e. genes which provided fitness benefits under competition with each of the tested organisms (Fig. 1). Interestingly, however, the set of genes for which loss of function resulted in fitness gains during competition was less consistent across the different organisms we used in this work (Fig. 2). Functional categories of genes that were particularly important to both gain and loss of fitness during competition were identified and are discussed below.

### Cell envelope modification genes are involved in fitness during competition

Many of the genes inferred to play a role in improved fitness during competition are involved in cell envelope biogenesis (Fig. 1). This includes a range of peptidoglycan biosynthesis genes, including: *murA*, encoding UDP-*N*-acetylglucosamine-1-carboxyvinyl transferase; PFL_5444, encoding a periplasmic ld-carboxypeptidase that catalyses the formation of 3-3 crosslinks in the peptidoglycan; *mrdA_2*, encoding a penicillin binding protein 2 with peptidoglycan transpeptidase activity; and *mviN*, encoding a membrane protein responsible for flipping lipid 2 to the outer leaflet of the inner membrane. It also includes genes involved in UDP-*N*-acetyl-α-d-glucosamine biosynthesis, an essential precursor for peptidoglycan and lipopolysaccharide biosynthesis, including: *glmM*, encoding phosphoglucosamine mutase; *glmS*, encoding l-glutamine:d-frucose-6-phosphate aminotransferase; *glmU*, encoding *N*-acetyl glucosamine-1-phosphate uridyltransferase/glucosamine-1-phosphate acetyltransferase; and *galU*, encoding UTP-glucose-1-phosphate uridyltransferase. It is likely that defects in peptidoglycan biogenesis would substantially increase Pf-5 susceptibility to a range of competition-related stressors, such as exposure to antimicrobial secondary metabolites and other toxic compounds.

Another aspect of cell envelope biogenesis that was observed to be important to fitness during competition was lipopolysaccharide biosynthesis. Important genes in this category include: PFL_0526, encoding an O-antigen polymerase; PFL_5646, encoding the lipopolysaccharide assembly protein LptD; *lpxB*, encoding a lipid A disaccharide synthase; and PFL_5102, encoding a glycosyltransferase. Loss of lipopolysaccharide in Gram-negative bacteria is known to cause enhanced susceptibility to antimicrobial compounds, which would explain why these genes are important during competition [33].

Two genes involved in folding and insertion of β-barrel proteins into the outer membrane of Pf-5 were found to be important during competition, these were *yaeT* and *yfgL*, encoding the BamA and BamB proteins, respectively, which are critical components of the BAM complex. Mutations in the BAM complex are known to cause a range of pleiotropic effects, including increased antimicrobial susceptibility, due to decreased abundance of a range of outer membrane proteins [34]. The *lnt* gene, encoding apolipoprotein *N*-acyltransferase, which mediates the final step in lipoprotein maturation, was also found to be important under competition conditions. This is also likely to result in pleiotropic effects on Pf-5, due to various lipoproteins not being properly localized. Similarly, the *tolA* gene, encoding the inner membrane component of the Tol–Pal system, which is important for outer membrane integrity and septal peptidoglycan processing during cell division, was in the set observed to play a significant role in Pf-5 fitness under competition conditions.

One of the driving forces in the evolution of the Gram-negative cell envelope is likely to have been coping with compounds secreted by competitor organisms in the environment. The importance of these cell envelope biogenesis genes for survival and persistence of Pf-5 when exposed to rhizosphere competitors provides some support for the hypothesis that many genes associated with clinical antibiotic resistance likely originate as defensive microbial competition genes in natural competitive environments such as soil [35]. The permeability barrier imposed by the Gram-negative cell envelope has now led to difficulties in developing new clinical antimicrobials for Gram-negative opportunistic pathogens, reflected in the World Health Organization category 1 pathogens entirely consisting of Gram-negative bacteria. Due to the intense competition of microbes in rhizosphere environments, there is potential for investigations into rhizosphere competitor interactions to identify novel specialized secondary metabolites that might overcome the Gram-negative envelope barrier.

### Genes for energy production and housekeeping functions affect fitness during competition

Energy production genes were also identified by TraDIS as important to fitness during competition (Fig. 1). Among the genes for which loss of function decreased fitness to a greater extent during competition was the tricarboxylic acid cycle (TCA) cycle gene *sucA*, which was observed to be linked to fitness during competition across all of the tested organisms. Genes *aceE* and ace*F* encoding components of pyruvate dehydrogenase that converts pyruvate to acetyl-CoA, which feeds into the TCA cycle, along with *pdhR*, encoding the pyruvate dehydrogenase complex repressor, were also important to Pf-5 fitness during competition with a subset of the tested organisms, particularly with the oomycete *Pythium spinosum*.

A large number of electron transport processes also appear to be linked to fitness under competition, including multiple subunits of NADH dehydrogenase (*nuoE*, *F*, *I*, *J*, *K*, *M*); however, for each of these genes, significant differences in fitness were again only observed in a subset of competition experiments. Fitness deficits during competition were also associated with loss of function in genes playing roles in aerobic respiration, particularly those involved in the use of cytochrome *c* as the terminal oxidase, including: *ccmB*, *C*, *D*, *F* genes, encoding components of cytochrome *c* biogenesis; cytochrome *c* oxidase (*ccoQ_1,ccoN_3*); cytochrome *c*4 (PFL_0084); several ubiquinone biosynthesis genes (*ubiB*, *D*, *H* and PFL_5965); genes encoding ubiquinol-cytochrome *c* reductase subunits (*petA*, *petB*, PFL_5078); and an electron-transferring-flavoprotein dehydrogenase gene (PFL_1818). Based on work in a range of other bacteria, it is likely this pathway is particularly important under low-oxygen conditions, and in *Pseudomonas aeruginosa* the CcoN_3 isoform of the core catalytic subunit has been shown to be important under anaerobic denitrification conditions and is thought to be particularly resistant to reactive nitrogen species [36]. The putative regulator PFL_0085 encodes a DNA-binding response regulator homologous to *roxR*, which has been shown to play a role in modulating the activity of a cyanide-insensitive cytochrome oxidase in *Pseudomonas aeruginosa* [37]. This gene contributed significantly to Pf-5 fitness during interactions with the same subset of organisms for which Pf-5 *ccoN_3* loss was significant (*Pythium spinosum*, *Pseudomonas* sp. Q2-87 and *Streptomyces bikiniensis *). This suggests that Pf-5 might be producing cyanide in response to competition with these three organisms, increasing the importance of the cyanide-insensitive cytochrome oxidase under these conditions.

There were also various genes involved in core cellular processes that were able to tolerate transposon insertions under control conditions, but not under the stress of competition, indicating increased importance of these genes during competition with a broad range of microbes. These included a small number of genes linked to transcription, translation and DNA replication, and a larger number associated with nucleotide biosynthesis and protein synthesis and fate (Fig. 1). Various purine and pyrimidine biosynthesis genes appear to be important for fitness competition with several of the tested competitors. These include the c*arA*, *carB*, *pyrB*, *pyrC* and *pyrE* genes, encoding four steps in the biosynthesis of pyrimidine nucleotides; the *dcd* gene, encoding dCTP deaminase in pyrimidine deoxyribonucleotide synthesis; *guaA*, *guaB*, *gmk* and *purH*, encoding four steps in the biosynthesis of purine nucleotides; and *cmk*, encoding cytidylate kinase in the pyrimidine salvage pathway. This is similar to what was observed in a previous TraDIS-based study, which identified *car*, *pyr*, *pur* and operons as important for survival in *Salmonella enterica* serovar Typhimurium during oral infection of livestock [38].

**Fig. 3.**
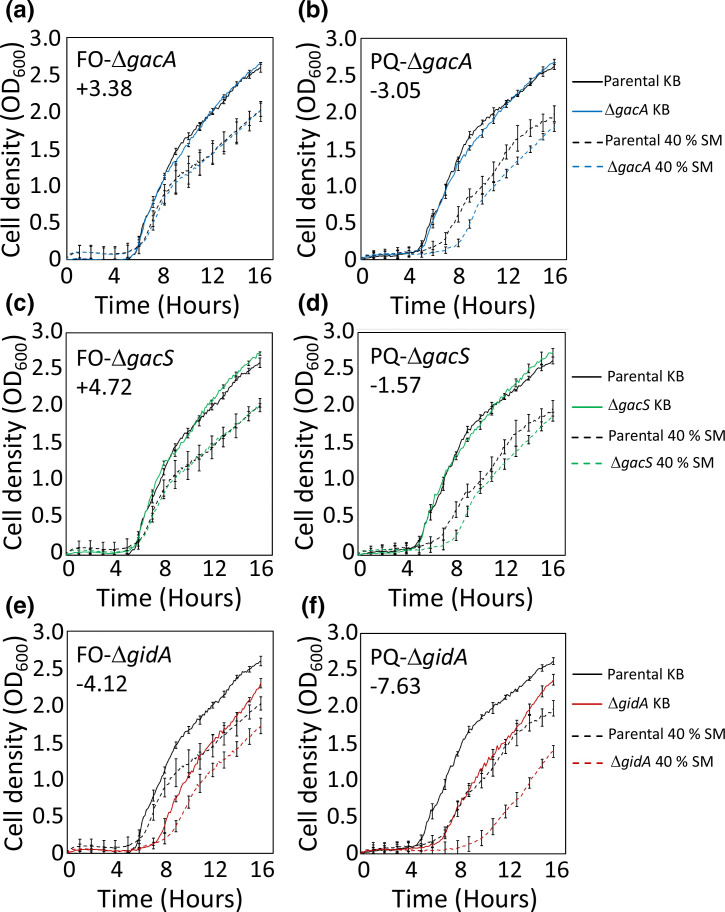
Growth of *Pseudomonas protegens* Pf-5 mutant strains in media containing secreted metabolites from *F. oxysporum* (FO) (left panels, **a, c and e**) and *Pseudomonas* sp. Q2-87 (PQ) (right panels, **b, d and f**). Spent media containing secreted metabolites was obtained from static cultures of the partner organisms and mixed 2 : 5 in fresh KB (40 % SM). The parental and mutant strains were grown in this media. For comparison, the parental and mutant Pf-5 strains were cultured in parallel in fresh KB alone. The optical density (at 600 nm) of cells grown at 30 °C was recorded for 16 h. The results of three replicate experiments are shown and error bars show the standard deviation. The log_2_-fold change values of corresponding mutant strains in the respective TraDIS experiments in shown as an inset in each plot.

A number of translation-related genes, including those encoding amino acid-tRNA ligases and tRNA-amino acid synthetases, as well as *trmE*, encoding a tRNA modification GTPase, and *infB*, encoding translation initiation factor IF-2, were found to be important for fitness during competition with a range of organisms. Also among this set was *gidA*, which encodes a subunit of the 5-carboxy methylamino-methyluridine tRNA synthase that modifies tRNAs at the wobble position. In *Escherichia coli, gidA* mutation strains exhibit moderate cell-division inhibition and it has been proposed the *gidA* gene may play a role in cell cycle regulation [39]. In this work, loss of *gidA* was observed to play a role in fitness during exposure to metabolites from all tested competitors, but was particularly detrimental in Pf-5 interactions with *Pseudomonas* sp. Q2-87 and *Streptomyces bikiniensis *, and to a lesser degree with *F. oxysporum*. To determine whether loss of *gidA* is important for dealing with competitor secreted factors, a *gidA* knockout mutation strain was cultured in spent media from cultures of *Pseudomonas* sp. Q2-87 and, for comparison, the fungal competitor *F. oxysporum* (Fig. 3a–f). While the *gidA* mutant strain showed an increased lag phase and impaired growth relative to wild-type Pf-5 in standard media, these growth defects were more pronounced in 40 % *F*. *oxysporum* spent media and especially in 40 % *Pseudomonas* sp. Q2-87 spent media. This is consistent with the TraDIS data and indicates that loss of *gidA* results in cells that are more sensitive to competitor metabolites, particularly those from certain soil bacteria, such as *Pseudomonas* sp. Q2-87.

Within the category of protein fate, three genes encoding components of the system responsible for periplasmic protein disulfide bond formation, *dsbA*, *dsbD* and *dsbE*, as well as *tatC*, encoding a component of the twin arginine-targeting protein translocase, and *yajC*, encoding a component of the Sec protein secretion system, were observed to contribute to fitness during exposure to competitors. This implies that periplasmic or secreted proteins play an important role in fitness in the presence of microbial competitors. The Lon protease, which plays important cellular roles in degradation of misfolded protein, and the DnaK chaperone protein were also both important for fitness in the presence of microbial competitors, suggesting one of the effects of secreted molecules from competitors is an increase in damaged proteins in Pf-5.

### Multiple regulatory genes, including *gacS* and *gacA*, are associated with fitness during competition

While many of the genes that confer fitness changes during competition were observed to be important across many of the tested competitors, there were subsets of genes which appear to be important for growth with only one or two specific partners or to play different roles in competitive fitness depending on the partner (Figs 1 and 2). This was the case for a number of regulatory genes identified as being associated with fitness during fungal, oomycete and bacterial challenge (Fig. 1). Among these were *gacS* and *gacA* that encode the membrane-bound sensor and response regulator, respectively, of a major two-component signal transduction system. Mutations in *gacS* and *gacA* were differentially selected depending on the competitor organism. Inactivation of *gacA* and *gacS* significantly reduced Pf-5 fitness in interactions with the related strain *Pseudomonas* sp. Q2-87 and with the oomycete *Pythium spinosum*, but resulted in significantly increased fitness in the competition experiment with the fungi *F. oxysporum* (Fig. 2). Mutations in *gacS* also increase Pf-5 fitness in competition experiments with *R. solani*, *G. graminis* var. *tritici* and the two *Streptomyces* species. The GacS/GacA system or close homologues have been shown to play central regulatory roles in many Gram-negative bacteria, controlling genes involved in interkingdom interactions through the Rsm pathway. In pathogens, GacS/GacA is responsible for inducing the expression of virulence genes [40, 41], whereas in biocontrol bacterial GacS/GacA is involved in inducing expression of biocontrol-associated secondary metabolite gene clusters and excreted enzymes [42, 43]. The signal(s) that lead to GacS activation have not been identified, but experiments performed in culture have shown that GacS activation occurs at high cell density, suggesting an endogenously produced signal [44].

Since the GacS/GacA system controls the production of numerous secreted factors, its inactivation can alleviate a large metabolic burden on the cell. Indeed, spontaneous *gacS* and *gacA* mutants show increased fitness, and are selected in fast-growing cultures of many *Pseudomonas* species under permissive growth conditions [45, 46]. These mutants are also likely to exist in natural environments, where they may act to enhance phenotypic heterogeneity within populations that could benefit the long-term survival of the lineage [47, 48]. However, when they are present within a population that requires the active production of secreted products, e.g. in active competition with a fungal competitor, *gac* mutant cells will benefit from public goods produced by their *gacS*+/*gacA*+ neighbours without the metabolic burden of producing them. In this situation, these cells are considered cheaters. Many studies have investigated the dynamics of cheater:non-cheater ratios for individual cell and population fitness in different conditions, including in the presence of antagonistic competitors [49–51]. The TraDIS experiments performed here offered additional insight into the importance of GacS/GacA in the presence of competitors. The initial ratio of *gac* mutants to *gacS*+/*gacA*+ cells in the TraDIS library was small [25], providing potential for cheating behaviour to be observed through an increase in *gac* mutant abundance. Fitness defects in *gac* mutants would also be detected by TraDIS as a reduction in *gac* mutant abundance.

The GacS/GacA regulon may play a unique role in interactions with closely related bacteria that have the potential for Gac signalling crosstalk. The TraDIS experiments showed lower fitness of both *gacS* and *gacA* mutant strains grown adjacent to *Pseudomonas* sp. Q2-87. *Pseudomonas* sp. Q2-87 encodes an orthologous *gacS*/*gacA* system to that encoded by Pf-5 and, thus, is likely to produce a related GacS activation signal. This may be linked to the importance of *gacS*/*gacA* during non-contact competition with *Pseudomonas* sp. Q2-87 compared to the other bacteria, *Bacillus* and *Streptomyces *, since the GacS activation signal produced by *Pseudomonas* sp. Q2-87 could facilitate crosstalk that increases the fitness of *gacS*+/*gacA*+ cells. To investigate this hypothesis, *gacS* and *gacA* mutant strain cells were cultured in media containing exudates from cultures of *Pseudomonas* sp. Q2-87 (Fig. 3b, d). There was an increased lag phase for the parental Pf-5 cells, and the *gacS* and *gacA* mutation strain cells when grown in 40 % *Pseudomonas* sp. Q2-87 spent media, which may be related to lower nutrient concentrations in this media, or due to the presence of antagonistic secondary metabolites (Fig. 3). However, the lag phase was noticeably longer in the *gacS* and *gacA* mutant strains, in line with the TraDIS results (Figs 1 and 2). The precise role of *gacS*/*gacA* in promoting cell fitness when grown in the presence of a related pseudomonad, or spent media from a related pseudomonad, will require considerable future investigation. However, since planktonic growth in spent media with no direct competitor organism is not likely to require extensive production of antimicrobial secreted products, the importance of GacS/GacA may be linked to its functions in controlling central metabolism, rather than secondary metabolism, possibly by promoting a shift in carbon metabolism that allows the cells to rapidly adapt to the available carbon sources in the media.

In contrast to *Pseudomonas* sp. Q2-87, the TraDIS experiments showed *gacS* and *gacA* mutants to have higher competitive fitness than *gacS*+/*gacA*+ cells when grown adjacent to *F. oxysporum* (Figs 1 and 2). To investigate this result further, we examined the growth rates of isolated *gac* mutants in medium containing secreted products from *F. oxysporum* (Fig. 3a, c). Growth in 40 % *F*. *oxysporum* spent media resulted in slower growth rates for both the parental Pf-5 strain and mutation strain growth profiles, which again may be related to nutrient depletion or presence of antagonistic secondary metabolites in this media. However, there was little difference in the growth of the parental strain and the *gac* mutants in this media, unlike in the TraDIS results, where *gacA* and *gacS* mutations conferred a fitness advantage over other mutations. As mentioned above, a major function of the GacS/GacA system is the control of secondary metabolite production and mutant strains produce little or no antifungal compounds [45]. The production of these compounds is energy and resource intensive. The *gacA* and *gacS* mutan may have cheated within the context of the TraDIS library, where they would still have benefitted from these antifungal metabolites produced by neighbouring *gacS*+/*gacA*+ cells that inhibit the growth of *F. oxysporum*, without the burden of producing these compounds themselves. This may explain their competitive advantage within this context.

Mutations in several regulatory elements involved in the *gacS/gacA* signal transduction cascade were also differentially selected during co-growth with some competitor organisms, including *rsmX* and *rsmA*. Upon activation by GacS, GacA activates expression of several small RNAs including *rsmX*. These small RNAs then act to relieve translational repression of numerous genes mediated by Rsm proteins, including RsmA. This in turn allows expression of several proteins, including enzymes involved in the production of secreted secondary metabolites, secreted enzymes and other regulators. Genes *rsmX* and *rsmA* were only strongly involved in fitness during exposure to *F. oxysporum*. These genes may also have a role to play more broadly in interactions with other competitors that was not observed here due to their functional redundancy or may be an artefact of the small size of these genes limiting the chance of transposon insertions.

CbrB, part of a two-component global *Pseudomonas* regulatory system that responds to carbon limiting conditions, was also found to influence fitness of Pf-5 during competition with a subset of our tested organisms. In *Pseudomonas aeruginosa *, a knockout in this system abolishes growth on arginine, histidine and polyamines as sole carbon sources [52]. This suggests the possibility that particular carbon sources might be preferentially important under some competitive conditions.

LexA, which controls the SOS response to DNA damage in many bacteria, appears to be important to Pf-5 fitness with two bacterial competitors, suggesting that these competitors may be producing secondary metabolites that have the capacity to result in DNA damage. Adjacent to *lexA* in Pf-5 is the gene *psrA*, which was important to Pf-5 fitness during competition with all tested organisms. There is some evidence to suggest in *Pseudomonas putida* WCS358 that PsrA may function as a positive regulator of *lexA* expression, which would be consistent with the SOS response being important for fitness under conditions of competition.

A number of RNA polymerase sigma factors and anti-anti-sigma factors were also observed to contribute to Pf-5 fitness during competition with specific partners. This included *rpoS*, encoding a homologue of the master regulator of the general stress response in *E. coli *; *rpoN*, encoding a RNA polymerase sigma-54 factor; PFL_0127, encoding an extracytoplasmic function (ECF) family RNA polymerase sigma-70 factor; and putative anti-anti-sigma factor PFL_1973. While the functions of these have not been elucidated in Pf-5, alternate sigma factors often play roles in responding to stress. MucA was also important to Pf-5 fitness in assays with *Pseudomonas* sp. Q2-87, the orthologue of this gene in *Pseudomonas aeruginosa* acts as an anti-sigma factor negatively regulating the alternative sigma factor *algU*, which is a positive regulator of alginate biosynthesis and a negative regulator of flagella-based motility.

### Loss of function of particular motility and cell-division genes leads to enhanced fitness during competition

Our assays revealed a set of genes for which loss of function resulted in significantly higher fitness during competition. In most instances, these fitness gains were specific to a subset of competitive interactions, with benefits often most pronounced in interactions with *F. oxysporum* (Fig. 2). Genes for which loss of function led to much stronger fitness effects during competition with *F. oxysporum* than the other tested competitors include PFL_1513, PFL_1514, and PFL_1515 encoding allophanate hydrolase subunits, and PFL_2244 (*N*-formylglutamate amidohydrolase), which are involved in amino acid metabolism, as well as the signal transduction genes *ptsP* and *ptsH*. The latter two genes are part of the phosphoenolpyruvate phosphotransferase system (PTS), a highly conserved phosphotransfer cascade in bacteria that mediates transport and phosphorylation of selected sugar substrates and is involved in regulating a range of cellular processes.

*Pseudomonas fluorescens* Q8r1-96 *ptsP* knockout mutation strains have been observed to show increased siderophore production and a less mucoid phenotype, as well as reduced production of the fungal inhibitor 2,4-diacetylphloroglucinol (2,4-DAPG) [53]. Based on this, *ptsP* loss in Pf-5 may be particularly beneficial during some fungal and bacterial interactions where the increased siderophore production associated with this gene loss assists in the specific competitive interactions, but is likely to be less favourable in interactions where 2,4-DAPG production is important (Fig. 2).

Mutations in genes associated with motility, particularly those associated with flagella biosynthesis, provided a selective advantage during exposure to metabolites from most of our tested organisms but not with the oomycete *Pythium spinosum*, indicating that loss of this structure was beneficial during competition with some fungi and bacterial species (Fig. 2). A possible explanation for this is that these complexes, which sit across the cell envelope, may provide entry sites for secreted metabolites from these particular competitors. Another possibility is that fitness advantages may occur through an altered biofilm production response, as flagella have also been shown to have functions in biofilm development [54]. In keeping with the involvement of flagella in competitive fitness, loss of two genes that are negative regulators of flagella synthesis/assembly resulted in significant decreased fitness. These genes were *fleN*, encoding a repressor of flagellar synthesis, and PFL_4484, encoding anti-sigma-28 factor FlgM, which negatively regulates flagellar gene expression (Fig. 1). The gene PFL_1134, encoding CheB1, part of a chemotaxis signal transduction system, also contributed to fitness during competition with a subset of the tested organisms, with loss of function providing a fitness advantage in experiments with two of the fungal and two of the bacterial competitors.

Loss of function of a set of cell-division-associated genes was also observed to result in significantly increased fitness during competition with some of the fungal and bacterial species tested (Fig. 2). This included genes *minC*, *minD* and *minE*, which make up the Min system responsible for ensuring cell division takes place at the centre of the dividing cell. Mutations in these are likely to lead to cells with broader than usual cell size distributions, based on work in other bacteria [55]. Also in this set was *ftsB*, encoding a key cell-division protein, with previous work in *E. coli* showing depletion of FtsB results in abnormal filamentous morphology [56]. These results suggest that some changes in cell size or morphology may confer an advantage to Pf-5 during exposure to some fungal and bacterial competitor metabolites. There is a variety of evidence in other bacteria that changes in cell morphology, particularly elongation, can lead to resistance or tolerance to antibiotics such as ciprofloxacin [57, 58]. Another possibility is that these mutations cause changes in cell permeability, affecting either uptake or efflux of secondary metabolites.

### Conclusions

There has been great interest in microbe–microbe competitive interactions in the context of biocontrol bacteria and the prevention of crop disease for many decades. Early research focussed on isolation of novel strains and characterization of the inhibitory compounds they produced. More recently, these organisms have been studied from a genetic and genomic standpoint, as well as from a transcriptomic one. Studies have mainly focussed on the antimicrobial products produced by these strains as determinants of a successful competitive phenotype; however, there is also growing interest and awareness of traits involved in persistence and adhesion [20]. The current study using TraDIS focussed on identifying genes that play a role in defence against the secondary metabolites produced by other competitive rhizosphere micro-organisms (Fig. 3). Information on defensive strategies used against soil microfauna may not only help us understand how microbes survive in competitive environments, but also give insights into the origins and evolution of antibiotic resistance. Some of the genes identified here as important for defence share similarities or are implicated in resistance to antimicrobials and antibiotics in related bacterial species, often through the modification of cell surfaces and membranes. Knowledge of defensive processes will also be useful in understanding successful persistence, competition and biocontrol by PGPR in agricultural settings.

**Fig. 4.**
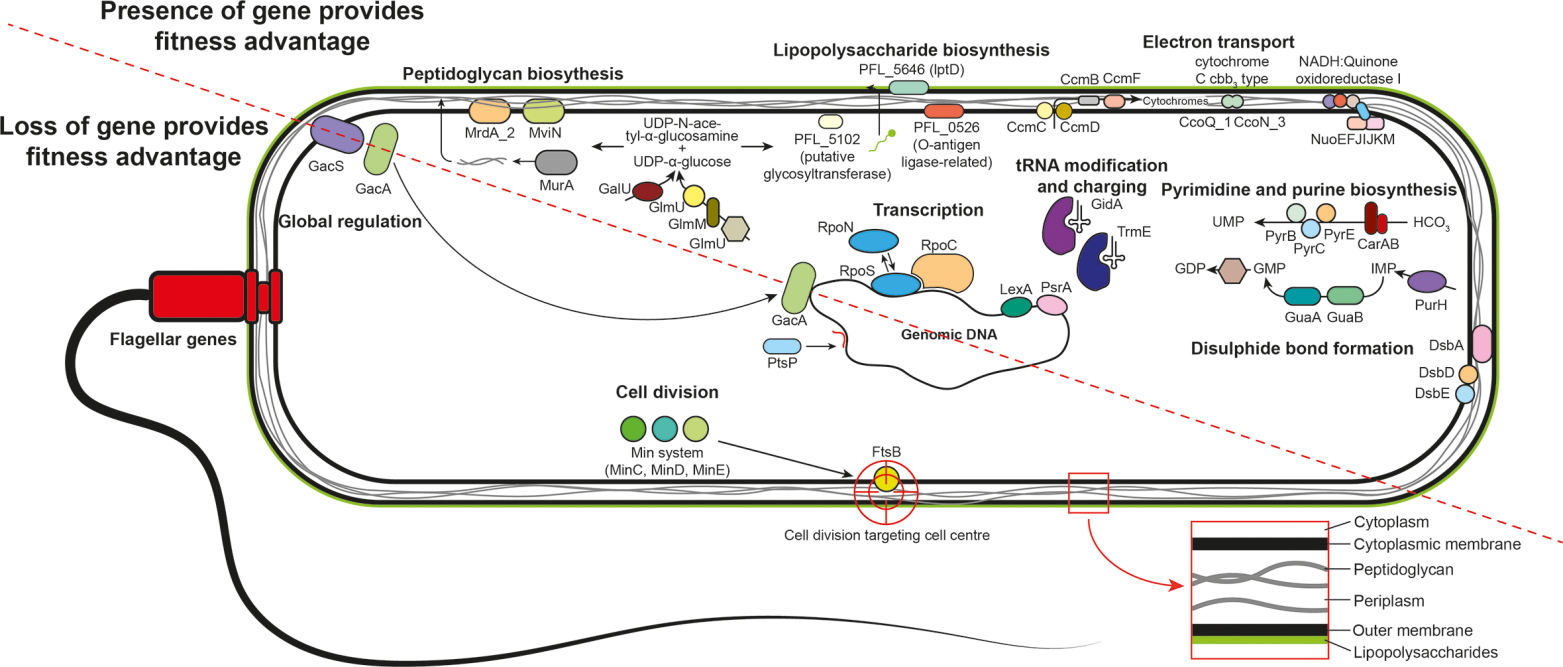
Conceptual diagram of the major genes and processes involved in *Pseudomonas protegens* Pf-5 defence from microbial competitor exudates.

The use of TraDIS has identified for the first time the set of genes affecting fitness of the PGPR Pf-5 during competition with diverse phytopathogens and other rhizosphere microbes. This has highlighted the importance of cell surface modification, cell division, energy production, nucleotide and protein synthesis genes, as well as several regulatory networks, for defence against microbial competitors by Pf-5 (Fig. 4). This knowledge expands our understanding of microbial competition from primarily offensive processes to defensive processes as well. Furthering our understanding of defence in microbe–microbe competition will contribute to applied microbiology undertakings, as well as improving our understanding of factors governing competitive outcomes in complex microbial communities.

## Supplementary Data

Supplementary material 1

Supplementary material 2
