# Supplementary material for: Delving into defence: identifying the Pseudomonas protegens Pf-5 gene suite involved in defence against secreted products of fungal, oomycete and bacterial rhizosphere competitors
Source: Microb Genom. 2021 Nov 17;7(11):000671. doi: 10.1099/mgen.0.000671 (PMC8743541; doi:10.1099/mgen.0.000671)
Supplement: Supplementary material 1 [file mgen-7-0671-s001.pdf]

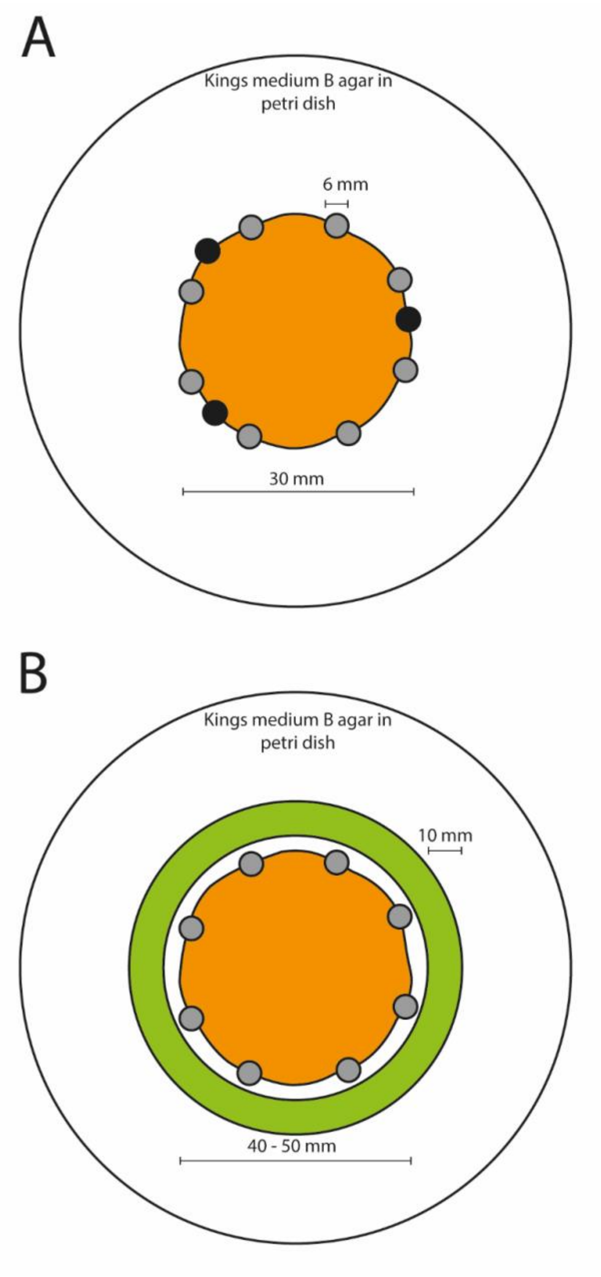

Figure S1. Diagrammatic representation of the layout for culturing of Pf-5 with secondary microbes on agar plates for HPLC analysis of metabolites (A) and TraDIS experiments (B). Growth of competitor microbes is shown as an orange region on the centre of the agar plates. Grey circles indicate the position of 6 mm diameter plugs taken from the agar plates in which WT Pf-5 cells were added to induce a competitive response. Black circles (A) indicate the position of the 6 mm plugs which were taken from the agar for HPLC analysis. The green ring (B) indicates the position of Pf-5 TraDIS library cells as they were placed on the agar plates in a ring around the growth of competitor microbes.

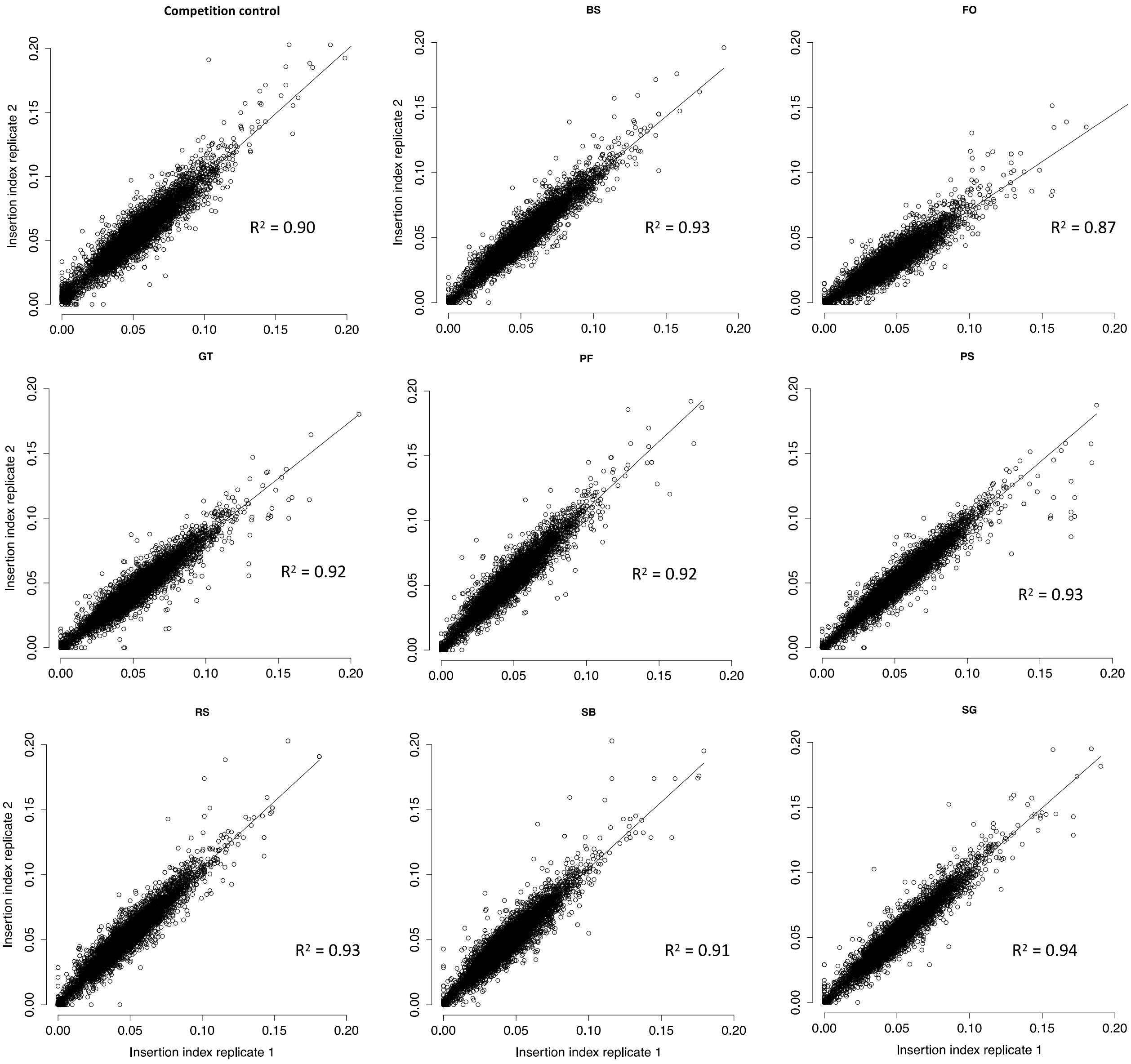

Figure S2. Correlation of gene insertion indexes for the two replicates of the *Pseudomonas protegens* Pf-5 mutant library for each fungal, bacterial or oomycete treatment*. Insertion index is calculated as the number of transposon insertion sites in a gene divided by the gene length. **Pythium spinosum* P.46.1 (PS), *Gaeumannomyces graminis* var. *tritici* (GT), *Fusarium oxysporum* (FO), *Rhizoctonia solani* (RS), *Bacillus subtilis* QST713 (BS), *Pseudomonas sp.* Q2-87 (PF), *Streptomyces bikiniensis* subspecies *bikiniensi* (SB) and *Streptomyces griseus.*

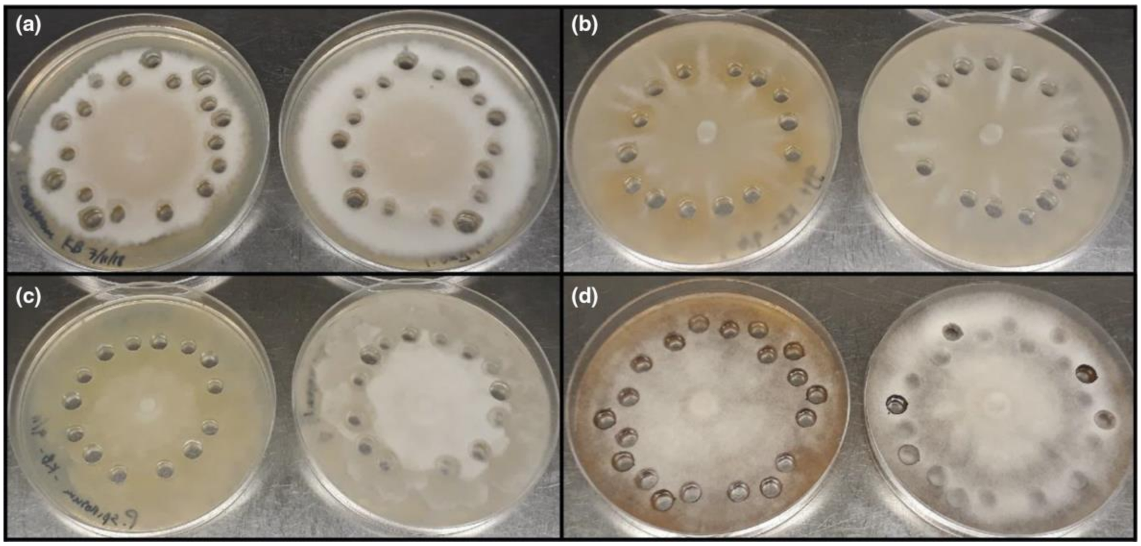

Figure S3. Examples of fungal and oomycete growth on Kings Medium B agar plates in the presence of live *Pseudomonas protegens* Pf-5 (left plate in each panel) and heat-treated killed *Pseudomonas protegens* Pf-5 (right plate in each panel). Plates are shown after two (b and c) or four (a and d) days of growth in the presence of Pf-5 which was placed in eight cored wells around the perimeter of the fungal or oomycete growth (20 ul of overnight culture per well). Fungal and oomycetes are *Fusarium oxysporum* (a), *Gaeumannomyces graminis var. tritici* (b), *Pythium spinosum* P.46.1 (c) and *Rhizoctonia solani* (d). Extra cored holes in the agar were taken for sampling and do not contain any additions.

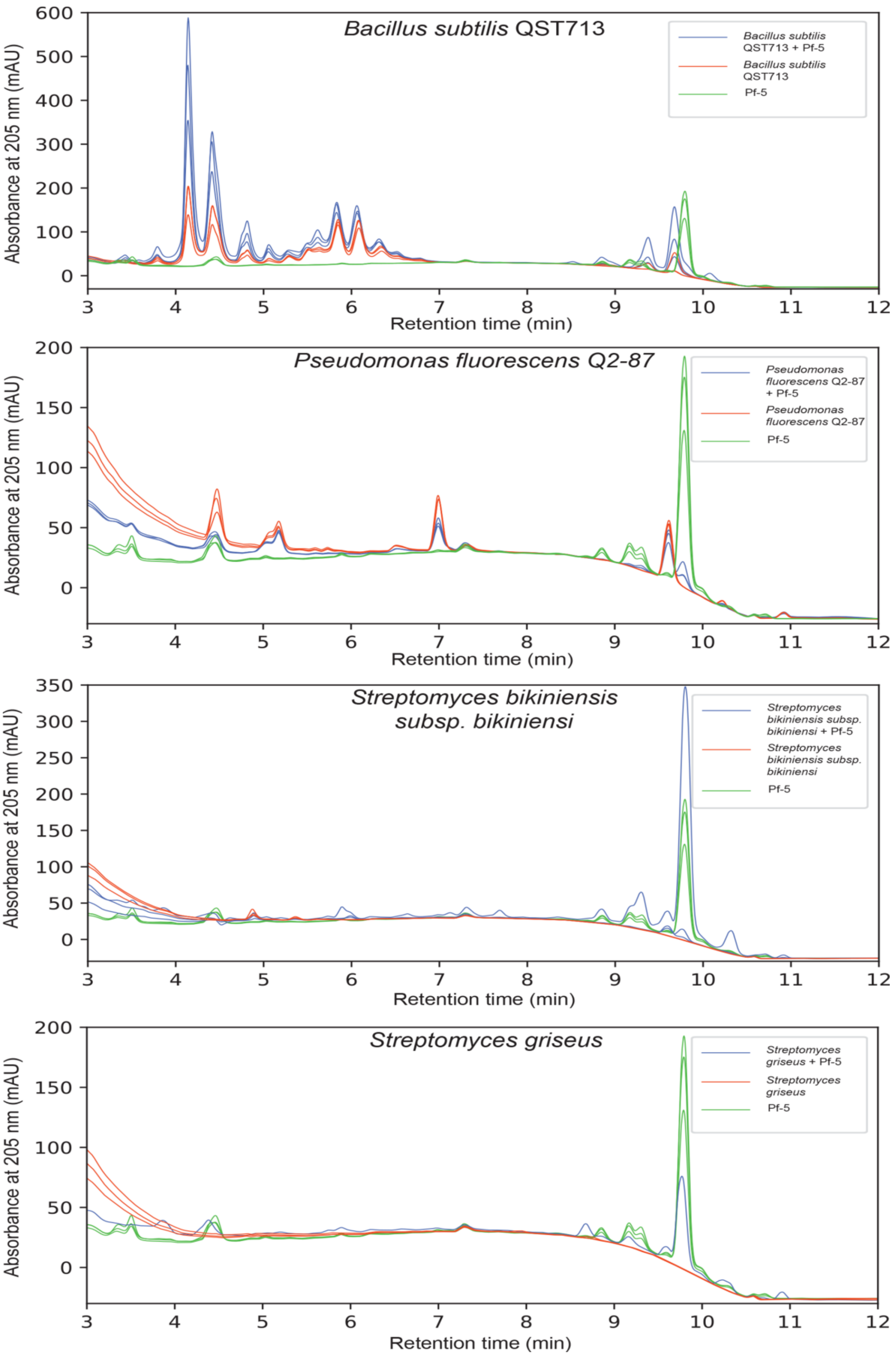
Bacillus subtilis QST713
Bacillus subtilis QST713 + Pf-5
Bacillus subtilis QST713
Pf-5
Absorbance at 205 nm (mAU)
Retention time (min)
Pseudomonas fluorescens Q2-87
Pseudomonas fluorescens Q2-87 + Pf-5
Pseudomonas fluorescens Q2-87
Pf-5
Absorbance at 205 nm (mAU)
Retention time (min)
Streptomyces bikiniensis subsp. bikiniensi
Streptomyces bikiniensis subsp. bikiniensi + Pf-5
Streptomyces bikiniensis subsp. bikiniensi
Pf-5
Absorbance at 205 nm (mAU)
Retention time (min)
Streptomyces griseus
Streptomyces griseus + Pf-5
Streptomyces griseus
Pf-5
Absorbance at 205 nm (mAU)
Retention time (min)

Figure S4. HPLC analysis of extracts from Kings medium B agar plates on which *Pseudomonas protegens* Pf-5 and bacterial competitors had been grown adjacent to each other for four days.

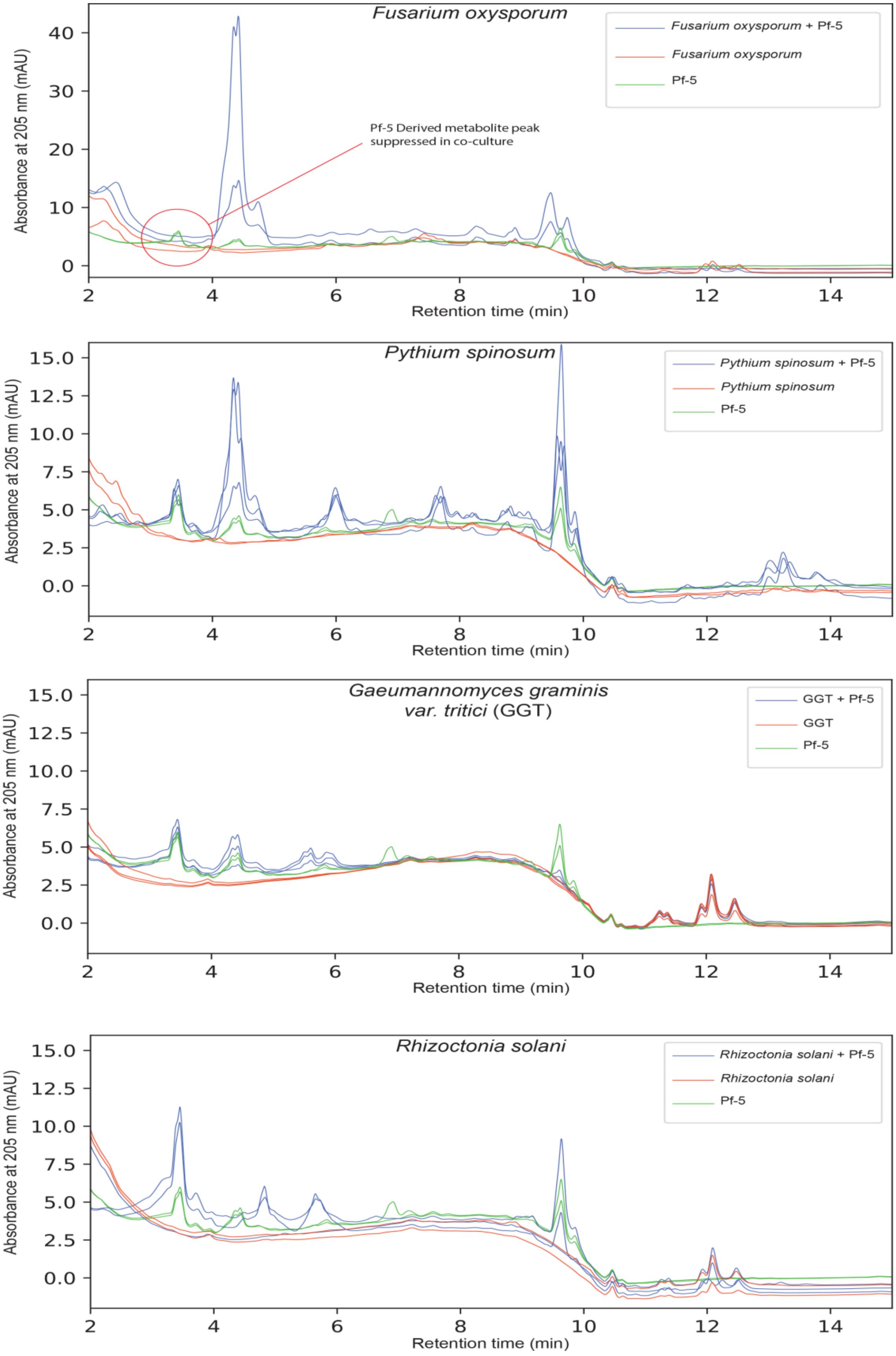
Fusarium oxysporum
Fusarium oxysporum + Pf-5
Fusarium oxysporum
Pf-5
Pf-5 Derived metabolite peak suppressed in co-culture
Absorbance at 205 nm (mAU)
Retention time (min)
Pythium spinosum
Pythium spinosum + Pf-5
Pythium spinosum
Pf-5
Absorbance at 205 nm (mAU)
Retention time (min)
Gaeumannomyces graminis var. tritici (GGT)
GGT + Pf-5
GGT
Pf-5
Absorbance at 205 nm (mAU)
Retention time (min)
Rhizoctonia solani
Rhizoctonia solani + Pf-5
Rhizoctonia solani
Pf-5
Absorbance at 205 nm (mAU)
Retention time (min)

Figure S5. HPLC analysis of extracts from Kings medium B agar plates on which *Pseudomonas protegens* Pf-5 and fungal or oomycete competitors had been grown adjacent to each other for three days.

Table S1. ***Pseudomonas protegens*** **Pf-5 transposon mutant library metrics from Bio-Tradis pipeline analysis. Species indicates the fungal, bacterial or oomycete species the Pf-5 mutant library was grown alongside.**

Species	Replicate	Total no. of reads	No. (%) of reads with matching transposon tag	No. (%) of aligned reads	No. of unique insertion sites	Average distance between unique insertion sites (bp)
Competition control	1	3,521,366	3,493,114 (99.2%)	2,122,933 (60.8%)	343,890	20.6
Competition control	2	4,758,755	4,712,581 (99.0%)	2,167,937 (46.0%)	360,273	19.6
Fusarium oxysporum	1	2,642,369	2,621,157 (99.2%)	2,101,066 (80.2%)	276,294	25.6
Fusarium oxysporum	2	2,655,603	2,634,749 (99.2%)	2,081,423 (79.0%)	192,766	36.7
Gaeumannomyces graminis var. *tritici*	1	2,792,201	2,772,558 (99.3%)	2,141,123 (77.2%)	325,128	21.8
Gaeumannomyces graminis var. *tritici*	2	1,718,549	1,701,172 (99.0%)	1,072,535 (63.0%)	283,618	24.9
Pythium spinosum P.46.1	1	3,252,994	3,224,845 (99.1%)	2,685,842 (83.3%)	331,752	21.3
Pythium spinosum P.46.1	2	1,813,018	1,797,481 (99.1%)	1,482,807 (82.5%)	326,497	21.7
Rhizoctonia solani	1	1,901,904	1,885,700 (99.1%)	1,523,032 (80.8%)	297,582	23.8
Rhizoctonia solani	2	3,450,805	3,425,670 (99.3%)	2,748,627 (80.2%)	322,297	22.0
Bacillus subtilis QST713	1	2,127,655	2,118,255 (99.6%)	2,050,590 (96.8%)	321,504	22.0
Bacillus subtilis QST713	2	2,830,068	2,817,579 (99.6%)	2,705,823 (96.0%)	316,736	22.3
Pseudomonas sp. Q2-87	1	2,076,259	2,067,020 (99.6%)	2,019,326 (97.7%)	286,924	24.7
Pseudomonas sp. Q2-87	2	3,362,868	3,347,914 (99.6%)	3,267,865 (97.6%)	323,028	21.9
Streptomyces bikiniensis subspecies *bikiniensi*	1	2,434,801	2,424,021 (99.6%)	2,367,645 (97.7%)	272,587	26.0

Streptomyces bikiniensis subspecies *bikiniensi*	2	3,296,639	3,282,155 (99.6%)	3,202,667 (97.6%)	298,110	23.7
Streptomyces griseus	1	2,114,048	2,104,480 (99.5%)	2,057,566 (97.8%)	320,503	22.1
Streptomyces griseus	2	2,414,029	2,403,225 (99.6%)	2,346,208 (97.6%)	328,174	21.6
